# Effect of Blend Levels on Composite Bread Prepared From Anchote (*Coccinia abyssinica*) Starch and Wheat Flour

**DOI:** 10.1155/ijfo/5705023

**Published:** 2025-07-23

**Authors:** Etalema Desta Tulu, Ramesh Duraisamy, Belay Haile Kebede, Alemu Mekonnen Tura

**Affiliations:** Department of Chemistry, College of Natural and Computational Sciences, Arba Minch University, Arba Minch, Ethiopia

**Keywords:** anchote, anti-nutritional, blending, composite flour, starch, wheat

## Abstract

Blending wheat flour with anchote starch offers a valuable approach to reducing the antinutritional factors present in anchote. However, the use of anchote starch as a blending ingredient for wheat bread has not yet been investigated. This study addresses this gap by extracting and characterizing starch from anchote (*Coccinia abyssinica*) as a supplementary ingredient to wheat flour bread. Anchote samples were collected from the western Oromia Region in Ethiopia, and wheat samples were obtained from the Bishoftu research center. Wheat flour was combined with anchote starch at varying levels of 5%, 10%, 15%, and 20%. The study analyzed proximate composition, mineral content, antinutritional factors, and functional properties for both anchote starch and the composite flour. Results, processed through one-way ANOVA at a 5% significance level, showed that moisture, ash, protein, fat, fiber, carbohydrate, and gross energy contents in the composite flour ranged, respectively, from 9.17% to 9.73%, 1.71% to 1.99%, 3.35% to 4.87%, 3.05% to 3.85%, 1.33% to 3.67%, 79.71% to 82.4%, and 370.49–374.71 kcal. As the proportion of anchote starch increased, mineral contents of calcium, sodium, iron, and zinc rose, while potassium, magnesium, and manganese levels declined. Functionally, increasing anchote starch led to higher bulk density and swelling power, while water and oil absorption capacities decreased. Antinutritional elements such as phytate, cyanide, and tannin were reduced with higher anchote starch levels. Sensory evaluations indicated that bread acceptability improved with increased anchote starch, reaching optimal levels at 20%. Overall, anchote starch shows promise for enhancing the nutritional profile of food products, adding beneficial minerals and fiber.

## 1. Introduction

Chronic and acute food insecurity remains a widespread issue in developing nations. Approximately 10% of developing countries face chronic food insecurity, a figure that can rise above 15% during frequent droughts, leading to acute food shortages [1]. Reducing poverty and hunger is a central goal of developing countries in order to address food insecurity; the approach has focused heavily on increasing cereal production. However, production gains have not proportionately lowered undernourishment rates due to a simultaneously growing population. This imbalance is further exacerbated by a farming system that relies on low-input, low-output, rain-fed agriculture and lacks diversification [2]. Another challenge in developing countries is reliance on imported cereals, particularly wheat, to mitigate food insecurity. Wheat (*Triticum aestivum* L.) is a staple crop globally and essential for producing baked goods due to its unique protein qualities. While some wheat types, such as *Triticum aestivum*, are suited for bread making, others like *Triticum durum* are ideal for biscuits and cookies [3]. However, importing wheat to meet domestic demand negatively impacts the balance of payments, straining the government's ability to fund essential imports, ultimately leading to foreign exchange shortages [4].

Therefore, it is important to design alternative methods that fully or partially replace cereal crops to ensure sustainable food security and market stability [5]. One of the indigenous Ethiopian root crops which can have the potential to partially substitute wheat is anchote. Anchote (*Coccinia abyssinica*) is one of the important root crops grown in the west, south, and southwestern regions of Ethiopia. It is a subsistence crop commonly produced to fill food security gaps during the hunger months (June to September) [6]. Nutritionally, anchote contains enough proteins, carbohydrates, Ca, Fe, and Zn. Medicinally, it is used for the treatment of gonorrhea, tuberculosis, cancer, malaria, and so forth. It also provides economic incomes due to the maximum yield from small-scale farms, thus used as a cash crop. The importance of anchote extends to social importance (served only during special ceremonies like thanksgiving day, weddings, betrothal, circumcision, birthdays, and “Meskel holiday”) [7].

The high-quality root crop anchote presents a viable alternative for partially replacing wheat flour in bread production. Bread is a staple food globally, rich in carbohydrates, vitamins, and minerals, making it a vital source of energy, dietary fiber, and essential nutrients like protein, B vitamins, and minerals—especially magnesium, calcium, and iron [8]. Using composite flours, where a portion of wheat flour is substituted with anchote flour, can reduce costs associated with importing wheat. However, as Demelash Hailu noted, using anchote flour directly in wheat flour substitution can result in high cyanide content, measuring up to 26.39 mg/100 g [8]. Since cyanide is water-soluble, extracting starch from anchote instead of using the whole flour helps lower cyanide levels. This study focused on blending wheat flour with anchote starch specifically to reduce antinutritional factors in anchote. The extracted anchote starch underwent physicochemical and proximate characterization and was then blended with wheat flour at levels of 5%, 10%, 15%, and 20%. The resulting composite flour was analyzed for proximate composition, mineral content, antinutritional factors, and functional properties, with all data evaluated using one-way ANOVA at a 5% significance level.

## 2. Materials and Methods

### 2.1. Sample Collection

Mature anchote roots were collected from Nekemte market, Oromia, Ethiopia. The sample was preserved in plastic bags and sent to the advanced chemistry lab at Arba Minch University, where it was kept in an ice box to prevent moisture loss. The sample was kept in case it was required once again. The Bishoftu Agricultural Research Centers provided the wheat.

### 2.2. Sample Preparation

#### 2.2.1. Wheat Flour Preparation

The wheat variety was cleaned by removing all extraneous materials. Wheat flour was obtained through the use of a grinder (PSAW, Model-133001, India). The wheat flour was passed through a sieve with a mesh size of 250 *μ*m to produce fine, homogenized flour. The wheat flour was stored at room temperature and wrapped in a polyethylene bag prior to use.

#### 2.2.2. Extraction of Starch From Anchote

A high-speed lab scale blender (BOXIYA, Model HJ-767/GS-PBJ-01, Germany) was used to quickly and thoroughly grind fresh anchote roots into 1-cm cubes. The sample was agitated for 5 min, suspended in 10% water, and filtered through Muslin cloth. The top liquid was decanted and thrown away after the starch in the filtrate had been allowed to settle for 2 h. The suspension was filtered through two layers of cheese cloth, and the filtrate was left to settle for 12 h. After discarding the supernatant, the sediment was twice washed with distilled water. The sediment was dried for 24 h at 45°C in a drying oven. The dried starch was pulverized and run through a 250-*μ*m filter before being placed in airtight plastic containers for further study [9–11].

#### 2.2.3. Composite Flour Preparation

Preliminary works were carried out for optimizing the blend ratio. The blend ratio was selected based on different compositions of anchote starch and wheat as shown in Table 1. Five different blending proportions were prepared [12].

### 2.3. Analysis of Anchote Starch and Composite Flour

#### 2.3.1. Proximate Analysis

The composite flour and anchote starch proximate analyses were performed using the AOAC (2000) technique. The following formula was used to calculate the carbohydrate content: 100 − (%crude protein + percent crude fiber + percent total ash + percent crude fat). The gross energy content was determined from the lipid, carbohydrate, and protein contents using conversion values of 4 kcal/g for protein, 9 kcal/g for fat, and 4 kcal/g for carbohydrates ([8, 12, 13], and [14]).

#### 2.3.2. Mineral Analysis

According to the AOAC (2005) standard, the mineral content was assessed using an atomic absorption spectrophotometer (BUCK Scientific, Model BUCK210, United States). After 1 g of the sample was reduced to ash, a known weight of ash was treated with 5 mL of 6 N HCl and dried on a hot plate. The solution was mixed with 15 mL of 3 N HCl and cooked on the hot plate until it boiled. A 50-mL graduated flask was filled with deionized water after the solution was chilled and filtered through Whatman No. 1 filter paper. Fe, Zn, Ca, Mg, K, Mn, and Na were determined. Both the samples and the standards had their absorbance measured. The mineral concentration of samples was determined from the standard calibration graph and was expressed in mg/100 g [8, 15].

#### 2.3.3. Antinutritional Analysis

Gilani et al. and Zelalem et al. [16, 17] proposed a method for determining phytate. The method developed by Holmes and Kennedy [18] was used to measure oxalate. The amount of tannin in the sample was quantified using the method published by Mitiku and Bora [8, 19]. Mitiku et al. [15] investigated the cyanide level of raw and differently treated flour samples.

#### 2.3.4. Functional Property Determination

The Onwuka method was used to measure the water absorption capacity (WAC), bulk density, swelling capacity, and oil absorption capacity (OAC) of the composite flour [20–22].

### 2.4. Preparation of Bread

Straight dough method was used to make the breads. Then, 1 kg flour (wheat flour and anchote starch), 5 g yeast, 5 g salt, and 900 mL water were used in the baking mix. All of the ingredients were blended by adding water, and after 15 min of kneading and shearing, nonsticky, homogeneous dough was produced. The dough was fermented in a fermentation cabinet for 60 min at 27°C. The dough was proofed for 20 min before being placed on an oil-greased pan and cooked for 15 min at 200°C. Finally, the bread was removed and allowed to cool for 1 h before the sensory examination [15, 23].

### 2.5. Sensory Evaluation of Bread

Using a nine-point hedonic scale, sensory qualities (taste, color, odor, texture, and overall acceptability) were evaluated. Then, 50 untrained consumers performed the sensory evaluation. The loaves of bread were served in test tins that had three-digit random numbers printed on them. After orientation, panelists were given three digit-coded samples in a random order, along with a cup of water to rinse their mouths between samples to prevent carryover bias. Analysis of the mean scores was carried out [8, 15].

### 2.6. Physical Properties of the Bread

The loaf volume of baked bread rolls was determined using the rapeseed displacement method. Each roll was cooled and measured 24 h after baking. The loaf was weighed using a digital balance to obtain its mass (gram). It was then placed in a box and covered with rapeseeds to measure volume (cubic meter). The displaced seed volume was recorded as the loaf volume. Specific volume was calculated as loaf volume (cubic meter) divided by loaf weight (gram).

 $$\begin{array}{c} \text{Specific volume}=\frac{\text{Loaf volume }\left({\text{cm}}^{3}\right)}{\text{Loaf weight }\left(\text{g}\right)}. \end{array}$$

### 2.7. Statistical Analysis

The proximate analysis, mineral analysis, and antinutritional analysis of five level blends of composite flour, as well as sensory evaluations of bread from each blend, were conducted using a completely randomized design. Wheat flour was blended with anchote starch at five different ratios: 100:0, 95:5, 90:10, 85:15, and 80:20 (shown in Table 1). The analysis was carried out, and the results were presented as the average standard deviation of three replicates [12]. The one-way ANOVA was used to analyze all of the generated data at a significance level of 5%. The study was performed with SAS 9.3 software (SAS, 2014).

## 3. Result and Discussions

### 3.1. Chemical Composition of Starch

The proximate composition of food is used to assess the nutritional potential of crops [24].  Table 2 displays the results of the percent yield and proximate composition of the starch recovered from anchote (*Coccinia abyssinica*).

It is evident from Table 2 that the percentage yield of starch derived from anchote was determined to be 70.41% ± 0.35%. This observed starch yield from anchote is comparatively lower than that previously reported by Getnet et al. The moisture content of the anchote starch analyzed in the current study was assessed to be 11.5% ± 0.5%. The moisture content serves as a direct indicator of the water content and an indirect indicator of the dry matter present in the samples [27]. A prior investigation [28] indicated that any sample with a moisture content below 14% possesses the ability to inhibit microbial proliferation, thereby ensuring enhanced storage stability. Moreover, the moisture content of starch samples ranging from 0% to 12% is deemed acceptable for both storage and subsequent processing, mitigating the risk of microbial contamination. As stated by Eze [27], low moisture content leads to the making of a more shelf-stable product. This specifies that the sample with moisture higher than 14% is not stable at room temperature and will support microbial growth, thereby generating flavors and smelly odors [27]. The minimal protein concentration (7.01%) was observed in the starch derived from anchote in the current investigation, in contrast to the protein levels found in starches extracted from potato and wheat (refer to Table 2). The protein concentration in any sample deemed necessary typically falls within the range of 7.0%–8.5% for the formulation of sweet biscuits [27]. Consequently, the anchote starch examined in this study, which is intended for partial substitution of wheat flour, possesses a protein content that is deemed adequate for the process of bread baking.

The lipid content present in anchote starch samples (0.69%) is comparatively minimal when compared with the lipid content found in starch derived from potato and wheat, as documented by Getnet et al. [9]. This phenomenon may be attributed to genetic factors, varietal differences, and the quality of the agricultural land, among other considerations; additionally, root and tuber plants primarily accumulate starch rather than lipids. Food substances characterized by low lipid content hold greater significance, as all food items containing lipids are indisputably prone to oxidative rancidity [20]. The crude fiber content of the starch was also evaluated and documented. The resultant measurement was 0.14%. This appears to represent the lowest quantification of crude fiber, which plays a crucial role in impeding the influx of glucose into the bloodstream and subsequently alleviating the colonic burden on the digestive apparatus, thereby diminishing the risk of colorectal malignancies in humans [29]. The obtained result was determined to be inferior to that previously reported by Sit et al. [10], who indicated a fiber content of approximately 0.21%.

The ash content of anchote starch derived from the current investigation was found to exceed that of wheat starch while remaining lower than that of potato starch. The ash content represents the residue following the combustion process, which corresponds to minerals (inorganic salts) originating from the fresh tuber, the application of fertilizers, and may also be attributed to soil and atmospheric contamination during processing [10]. According to Iwe et al. [20], the ash content of a food sample generally reveals the mineral composition (elements) inherent to that particular food. Based on recommendations from the World Health Organization, a total ash content of 5% is advised for weaning foods [25]. Therefore, the ash content of the analyzed anchote starch falls within the permissible limits and additionally suggests the potential for fortification with other food items to enhance the ash content. The carbohydrate composition of the starch sample flour was quantified to be 79.60% ± 0.85% (refer to Table 2). The findings indicated that the starch extracted from anchote exhibited the highest carbohydrate concentration. Typically, existing literature indicates that the starch derived from fresh anchote encompasses a range of 75%–90% in terms of carbohydrate content. Within this context, starch is regarded as the predominant constituent of the anchote root, succeeded by various sugars including sucrose, glucose, fructose, and maltose. In the current investigation, the carbohydrate content within anchote starch was observed to be even more elevated. This elevated carbohydrate concentration renders it an excellent energy source [26].

### 3.2. Proximate Analysis of Composite Flour

Table 3 shows the composite flour's proximate composition (moisture content, ash, protein, fat, fiber, carbohydrate, and energy).

#### 3.2.1. Moisture Content

The observed values of the moisture content within the composite flour exhibited no statistically significant variation (*p* < 0.05) attributable to differing blending ratios, with measurements recorded at 9.17 ± 0.29, 9.50 ± 0.50, 9.73 ± 0.25, 9.55 ± 0.33, and 9.48 ± 0.03 for flour samples designated as WAS_0_, WAS_1_, WAS_2_, WAS_3_, and WAS_4_, respectively, (Table 3). Findings from the current investigation indicated that the moisture content of the composite flour was inferior to that of wheat flour incorporated with cassava starch, which was quantified at 9.98% ± 0.03% as documented by Ajatta et al. [30]. Lagnika et al. [31] have posited that diminished moisture levels exert a favorable influence on the extended shelf life of composite breads by inhibiting the proliferation of microbial entities responsible for spoilage. The regulation of moisture content in food items and consumable goods is of paramount importance, as elevated moisture levels can significantly compromise the product's shelf life [31].

#### 3.2.2. Ash Content

The ash content values of the flour samples labeled as WAS_0_, WAS_1_, WAS_2_, WAS_3_, and WAS_4_ were determined to be 1.97% ± 0.01%, 1.99% ± 0.02%, 1.99% ± 0.01%, 1.93% ± 0.01%, and 1.71% ± 0.04%, respectively, as presented in Table 3. The findings regarding ash content in the current investigation revealed that an increase in the partial substitution of wheat flour with anchote starch was concomitantly associated with a gradual decrease in the ash content of the composite flour. The samples WAS_1_ and WAS_2_, each exhibiting an ash content of 1.99%, contained the highest total ash levels, while WAS_4_, with an ash content of 1.71%, exhibited the lowest. Consequently, the samples characterized by elevated percentages of ash content are posited to possess substantial quantities of mineral elements, which are beneficial for enhancing metabolic processes as well as promoting growth and development [31]. According to the recommendations set forth by World Health Organization (WHO)/ Food and Agriculture Organization (FAO) in 2004, the ash content of food items should not exceed five; notably, all composite samples analyzed in the present study adhered to these established standards [32].

#### 3.2.3. Crude Fat

From the present result, the crude fat content of WAS_1_ differed significantly at *p* < 0.05 from the rest of the samples and decreased after the addition of anchote starch at different proportions. The values were found to be 3.85%, 3.98%, 3.76%, 3.62%, and 3.05% for WAS_0_, WAS_1_, WAS_2_, WAS_3_, and WAS_4_, respectively. The highest fat content (3.98% ± 0.03%) was found for composite flour containing WAS_1_ anchote starch. On the other hand, composite flour from WAS_4_ of anchote starch contains the lowest (3.05%) fat content. A comparable trend was reported by Ajatta et al. [30] in which increasing the quantity of cassava starch in the composite flour can decrease the crude fat content of the final products. Lagnika et al. [31] described that the fat content of composite should be ranged from 10% to 25%, in which the result of the present study is less than the daily recommended fat content [31, 32].

#### 3.2.4. Crude Fiber

The crude fiber content of the composite flour decreased as more wheat flour was replaced with anchote starch. Specifically, the fiber content was recorded as 3.67% ± 0.03%, 3.33% ± 0.06%, 3.02% ± 0.07%, 2.16% ± 0.17%, and 1.33% ± 0.03% for WAS_0_, WAS_1_, WAS_2_, WAS_3_, and WAS_4_, respectively. This decline can be attributed to the low fiber content of anchote starch (Table 3). As reported by Abebe et al., wheat flour contains 0.6%–1.9% insoluble fiber and 0.1%–2.8% soluble fiber, resulting in a total fiber content of 0.7%–4.7% [12]. Except for the WAS_4_ formulation, the crude fiber content of the composite flours remained above 1.5%, which complies with the maximum permissible fiber content in flour specified by Gemede and Fekadu [32]. Higher crude fiber levels are beneficial for managing conditions such as obesity, diabetes, cancer, gastrointestinal disorders, and colon cancer prevention [32]. The WHO and the FAO recommend a daily fiber intake of less than 5%, and all the composite flours formulated in this study comply with this standard [30, 32].

#### 3.2.5. Protein Content

The study's results showed the blending ratio had a minor, nonsignificant effect (*p* < 0.05) on the composite flour's crude protein content. The highest protein content (4.87%) was found in WAS_0_, while the lowest (3.35%) was observed in WAS_4_. The decline in protein content with increasing proportions of anchote starch is likely due to its low protein levels. These findings are consistent with those of Mitiku [8], who reported a reduction in the protein content of snacks when breadfruit flour was supplemented with starch-based products.

#### 3.2.6. Carbohydrate Content

The carbohydrate content of the composite flours increased with higher levels of anchote starch substitution. The highest carbohydrate content (82.40%) was found in WAS_4_ (80% wheat flour and 20% anchote starch), while the lowest (79.71%) was recorded in WAS_1_ (95% wheat flour and 5% anchote starch). The high carbohydrate content in WAS_4_ is attributed to the naturally high carbohydrate levels in anchote starch [8, 15]. Compared to the UICEC/WHO [33] standards, the carbohydrate content in the flours from this study exceeds the minimum regulatory requirement of 37% [31, 32].

#### 3.2.7. Energy Content

The results of the study indicated that the blending ratio did not significantly affect (*p* < 0.05) the energy value of the composite flours. However, the energy content decreased as the proportion of anchote starch increased. The highest energy value (374.71 kcal) was observed in WAS_0_ (100% wheat flour), while the lowest (370.49 kcal) was recorded in WAS_4_ (80% wheat flour and 20% anchote starch). The lower energy values can be attributed to the low-fat content of both wheat flour and anchote starch. The variation in energy content is linked to differences in their protein, fat, and carbohydrate compositions. A similar trend was reported by Ashogbon and Akintayo [34] in a study on fortifying wheat flours with plantain and soybean flour.

### 3.3. Functional Properties of Composite Flour

The functional characteristics of a food material affect its application and ultimate use [35], as well as how it interacts with other food components.  Table 4 displays the functional properties of blended wheat and anchote starch.

#### 3.3.1. WAC

From Table 4, the WAC of all the flours ranged from 107% to 146%. The WAC values of the composite flours from WAS_1_, WAS_2_, and WAS_3_ did not differ significantly from each other (*p* > 0.05), but they were significantly different (*p* < 0.05) from WAS_0_ (100% wheat flour) and WAS_4_ (80% wheat flour and 20% anchote starch). The highest WAC of 146% was observed in WAS_0_, while the lowest WAC of 107% was in WAS4. The findings of this study suggest that adding anchote starch to wheat flour affects the WAC. Similar observations were made by Kaushal et al. [36]. Suresh Chandra and Samsher [37] noted that lower WAC in some flours could be due to a reduced presence of polar amino acids. The decrease in WAC after incorporating anchote starch may be attributed to increased amylose leaching and solubility, which disrupts the crystalline structure of the starch. Flours with higher water absorption tend to have more hydrophilic components like polysaccharides [38]. Overall, the results indicate that higher supplementation of anchote starch reduces the WAC of the flour samples.

#### 3.3.2. OAC

OAC refers to the ability of the flour mix proteins to absorb and retain oil, which affects the texture and mouthfeel of food products [37]. According to Table 4, the OAC ranged from 104% to 146% across all the composite flours. The composite flour WAS_0_ had the highest OAC at 147.00% ± 4.00%, while the lowest OAC was observed in WAS_4_ at 104.67% ± 4.16%. It is evident that the OAC of the composite flours decreased as the proportion of anchote starch increased. The reduction in OAC after adding anchote starch is likely due to differences in the presence of nonpolar side chains, which could bind to the hydrocarbon side chains of the oil in the flours [35]. Additionally, the higher moisture content in the wheat and anchote starch may reduce the OAC, as the pores in the flour might be occupied by water [36].

#### 3.3.3. Bulk Density

Bulk density is an important factor in determining packaging requirements and material handling [39]. In this study, the bulk densities of the composite flours ranged from 0.596 to 0.627 g/cm^3^ (Table 4). The highest bulk density (0.627 g/cm^3^) was observed in the WAS_3_ flour, while the lowest (0.596 g/cm^3^) was for wheat flour (WAS_0_). The bulk density of the flour samples increased with a 15% substitution of anchote starch for wheat flour but decreased when 20% anchote starch was used. The study showed that reducing the proportion of wheat flour increased the bulk density of the composite flours, which aligns with findings from Eltayeb et al. [39]. A high bulk density in flour suggests its suitability for use in bread preparations, while low bulk density is advantageous for formulating complementary foods [40]. From a nutritional perspective, low bulk density is beneficial because it encourages the consumption of a larger quantity of the lighter food, which results in increased nutrient intake for the consumer [39].

#### 3.3.4. Swelling Power

The swelling capacity of the flour blends ranged from 2.56 to 3.92 g/g (Table 4). According to Table 4, the sample WAS_4_ was significantly different from the other flour samples (*p* < 0.05) and had the highest swelling capacity at 3.92 g/g. Samples WAS_1_ (2.90 g/g) and WAS_2_ (2.03 g/g) were also significantly different from each other, with swelling power increasing as the amount of anchote starch in the composite flour increased. Offia-Olua et al. [38] noted that the swelling capacity of flour reflects the degree of associative forces within the granule. The higher swelling capacity of the wheat flour may be attributed to its greater carbohydrate and protein content, as well as its higher WAC. An increase in WAC typically leads to higher swelling power [40].

### 3.4. Mineral Content of Composite Flour

Effect of blending ratio of wheat and anchote starch on mineral contents of composite flour was shown in Table 5.

The mineral contents of samples analyzed in the present study ranged from 2.48 to 6.67, 5.83 to 5.39, 0.14 to 2.37, 0.24 to 2.59, 0.45 to 2.67, 0.68 to 0.42, and 3.02 to 1.83 mg/1 g for calcium, magnesium, zinc, iron, sodium, manganese, and potassium, respectively, as the content of anchote starch increases from 0% to 20% in the blended mixture. The variation observed could be due to the compositional difference in terms of mineral content between the crops used in the blends. For calcium, zinc, iron, and sodium, an increasing trend was observed with increasing anchote starch. Meanwhile, magnesium, manganese, and potassium amounts of the flour decreased as the amount of anchote starch increased.

#### 3.4.1. Calcium

From the present result, the calcium content of flour samples varied between 2.48 and 6.67 mg/1 g (Table 5). The calcium content of the flour samples investigated in the present study was significantly affected (*p* < 0.05) by the addition of anchote starch. The highest (6.67 mg/1 g) result was obtained from WAS_4_ (20% anchote starch and 80% wheat flour), while the lowest (2.48 mg/1 g) identified in flour WAS_0_ (100% wheat flour). The calcium content of flour increased with the increment of some anchote starch proportion in the mixed flour sample. The trend showed that there was an increment in the amount of calcium as the amount of anchote starch increased in the blend. Higher Ca content in the present study found in WAS_4_ flour may be attributed to a higher concentration of calcium in the anchote root. Calcium is the most common mineral in our body and is indispensable for the strength of the skeleton and hardness of teeth. It also plays numerous functions in the body [40].

#### 3.4.2. Zinc

In the present study, the zinc content of flour samples was varied from 0.14 to 2.37 mg/1 g (Table 5). The zinc content of the flour samples investigated in the present study was significantly affected by the addition of anchote starch. There were no significant differences observed among WAS_0_, WAS_1_, and WAS_3_ composite flour at *p* < 0.05 for zinc contents. The results also revealed that an increased Zn content was observed when there was a high concentration of anchote starch in the composite flour. Higher mineral content in the present study found in different flours may be attributed to a higher concentration of calcium and zinc in the anchote starch used for supplementation of composite flours [41].

#### 3.4.3. Iron

The iron content of flour samples was varied between 0.24 and 2.59 mg/1 g (Table 5). The highest Fe content was determined in flour prepared from WAS_4_ (80% wheat flour and 20% anchote starch) while the lowest result value of Fe was reported for flour from WAS_0_ (100% wheat flour. This might be due to an increase in the proportion of anchote starch. The high iron content increases the hemoglobin level of the blood that helps more oxygen to be transmitted [42].

#### 3.4.4. Sodium

In the present study, the content of sodium in the composite flour ranged from 0.45 ± 0.00 to 2.67 ± 0.13 (Table 5). The highest Na content was determined in flour prepared from WAS_4_ (80% wheat flour and 20% anchote starch) while the lowest value of Na was reported for flour from WAS_0_ (100% wheat flour). The Na content of flour increased with the increment of some anchote starch proportion in the mixed flour sample. However, the result of the present study is in line with the concentration of sodium found in food given by American Guidelines. According to the Dietary Guidelines for Americans, diets higher in sodium are associated with an increased risk of developing high blood pressure, which is a major cause of stroke and heart disease [33, 42].

#### 3.4.5. Magnesium

From the present study, the concentration of Mg in the composite flours ranged between 5.39 ± 0.10 and 5.83 ± 0.10 mg/1 g (Table 5). The Mg content of the flour samples investigated in the present study was significantly affected by the supplementation of anchote starch. The highest Mg content determined in flour was prepared from WAS_0_, and the lowest result value of Mg was reported for flour from WAS_4_. However, when compared with other root and tuber crops, the Mg concentration of wheat flour blended with anchote starch decreases as the concentration of starch increases.

#### 3.4.6. Manganese

From the present study, the manganese content of the composite flour ranged from 0.42 ± 0.00 to 0.68 ± 0.05 mg/1 g (Table 5). The highest Mn content was determined in composite flour prepared from WAS_0_, and the lowest result value of Mn was reported for flour from WAS_4_. The results also revealed that a decreased Mn content was observed when there was a high concentration of anchote starch in the composite flour. But consumption of foods rich in micronutrients like Mn helps in building a strong immune system, thereby helping the body to absorb, utilize, and digest nutrients [43].

#### 3.4.7. Potassium

From the present study, the potassium content of the composite flour ranged from 1.83 ± 0.09 to 3.02 ± 0.18 mg/1 g (Table 5). The highest K content was determined in composite flour prepared from WAS_0_, and the lowest result value of K was reported for flour from WAS_4_. The results also revealed that a decreased K content was observed when there was a high concentration of anchote starch in the composite flour. Based on a previous study, high potassium in humans plays a protective role against hypertension, stroke, cardiac dysfunctions, renal damage, hypercalciuria, kidney stones, and osteoporosis [43].

### 3.5. Antinutritional Factors

Table 6 shows the antinutritional constituent of the wheat flours and anchote starch (control and composite flour). This study shows the level of toxic substances in the samples.

#### 3.5.1. Phytate Content

The study found that the phytate content of the composite flour decreased as the proportion of wheat flour replaced with anchote starch increased. The phytate content was measured at 1.23 mg/g in the control sample (WAS_0_) and reduced to 1.16, 1.18, 1.04, and 0.92 mg/g in WAS_1_, WAS_2_, WAS_3_, and WAS_4_, respectively. This decline can be attributed to the lower phytate levels in anchote starch. The highest phytate content was observed in WAS_0_, while the lowest was in WAS_4_. The phytate concentrations in this study were below the permissible range. In contrast, diets primarily based on vegetarian staples or in rural areas of low-income countries often average 2000–2600 mg of phytates daily [41]. Although the phytate levels in these flours are low, they may still bind essential minerals like calcium, magnesium, iron, and zinc, reducing their bioavailability. High phytate intake can negatively impact health by forming insoluble complexes with minerals, decreasing the absorption of calcium, magnesium, iron, and phosphorus. This is primarily due to the higher phytate content in wheat flour [44].

#### 3.5.2. Condensed Tannin

Condensed tannins are considered antinutritional because they form insoluble complexes with digestive enzymes and hinder iron absorption. In this study, the tannin content in the flours ranged from 0.03 mg/g to not detectable (ND). The highest tannin concentration (0.03 mg/g) was recorded in WAS_0_, while tannins were undetectable in WAS_1_, WAS_2_, WAS_3_, and WAS_4_ (Table 6). These findings indicate that increasing the proportion of anchote starch does not elevate the tannin content in cooked foods. As noted by Abebe et al. [12], tannins can impair protein digestion and reduce mineral bioavailability, particularly iron and calcium. However, given the low total daily tolerance for tannic acid, the toxic effects of tannins at these levels are unlikely to be significant.

#### 3.5.3. Oxalate Content

Table 6 presents the oxalate concentrations in composite flours made from wheat flour and anchote starch. The oxalate content increased with higher levels of anchote starch supplementation due to its naturally higher oxalate levels. The oxalate content was measured as 2.20, 11.00, 13.93, 18.33, and 22.00 mg/g for WAS_0_, WAS_1_, WAS_2_, WAS_3_, and WAS_4_, respectively (Table 6). These values are significantly lower than those reported for raw taro (156.33 mg/100 g) and raw anchote (6.56 mg/100 g) by Alcantara et al. [45] and Gemede and Fekadu [32]. Dietary oxalates have been associated with reduced mineral bioavailability, particularly of divalent minerals like calcium and magnesium, and may contribute to conditions such as rickets [44]. Patients are advised to limit their daily oxalate intake to less than 50–60 mg [21]. Compared to these guidelines, the oxalate content in the flours analyzed in this study is relatively low. Employing processing methods such as starch extraction before consuming anchote tubers could enhance mineral bioavailability and positively impact consumer health [16].

#### 3.5.4. Cyanide Content

Table 6 highlights the cyanide concentrations in composite flours made from wheat flour and anchote starch. The cyanide content increased with higher levels of anchote starch supplementation due to its naturally higher cyanide concentration. The highest cyanide level (3.63 mg/g) was observed in the WAS_4_ formulation (20% anchote starch), while the lowest (3.16 mg/g) was found in 100% wheat flour. There were significant differences (*p* < 0.05) between the cyanide content of WAS4 and the control sample. However, the cyanide levels in this study are much lower than those reported by Mitiku [8], who found 26.39 mg/g of cyanide in bread prepared with 20% anchote flour supplementation. The cyanide concentrations in the current study are well below the toxic threshold of 50–200 mg, indicating that anchote starch is safe concerning cyanide poisoning [45]. While food crops provide essential nutrients, they may also contain antinutritional factors, such as cyanogenic glucosides, which can negatively impact health by reducing protein digestion, growth, and the absorption of iron and zinc [45].

### 3.6. Physical Characteristics of Bread Samples

The physical characteristics data of the breads produced from flours of wheat and anchote starch, which included loaf weight, loaf volume, and specific volume, were presented in Table 7. The loaf weight values of breads produced with different proportions of anchote starch added exhibited significant (*p* < 0.05) differences among themselves and from that of 100% wheat bread. The values ranged from 81.20 g of the 100% wheat bread to 92.83 g of the bread with 20% anchote starch addition, with significant differences among them, except between breads containing 5%, 10%, and 15% anchote starch. The loaf weight increased with an increase in anchote starch proportion. Mitiku [8] identified the quantity of dough baked, along with its moisture content, as the primary determinants of loaf weight. During baking, carbon dioxide diffused out of the dough. Increased moisture absorption and reduced air entrapment could account for the greater loaf weight, leading to a denser dough and consequently heavier loaves.

The loaf volume data presented in Table 7 show significant differences (*p* < 0.05) among bread samples with varying levels of anchote starch. The highest loaf volume (360.88 cm^3^) was recorded for 100% wheat bread, while the lowest volume (291.67 cm^3^) was observed in bread containing 20% anchote starch. This reduction in loaf volume with increasing anchote starch is likely due to the decreased gluten content, as lower wheat proportions reduce the formation of carbon dioxide bubbles within the dough. Gluten in wheat flour is essential for trapping carbon dioxide gas, creating a network of bubbles that give bread its spongy texture and large volume. A smaller loaf volume indicates a denser, more compact crumb structure [8]. The reduction in loaf volume with the inclusion of non-wheat flours, such as anchote starch, has been previously attributed to the dilution of gluten in composite flours [8, 31]. Table 7 also highlights the specific volume of the breads, showing significant differences (*p* < 0.05) between samples with varying anchote starch levels. 100% wheat bread had the highest specific volume (4.44 cm^3^/g), while the lowest specific volume (4.14 cm^3^/g) was recorded for bread with 20% anchote starch. The decrease in specific volume is linked to the reduced loaf volume and increased loaf weight in breads containing anchote starch. Specific volume reflects the relationship between loaf weight and volume, which is influenced by the dough's rising capacity during baking [8, 32].

### 3.7. Sensory Evaluation

Sensory evaluation (color, taste, odor, and texture) of breads produced by blending wheat with anchote starch was done by 50 untrained panelists (28 men and 22 women) and their results are presented in the table below depending on the type of parameters.

#### 3.7.1. Color

Color is an important qualitative characteristic that seems to play a significant role in customers' initial acceptance of baked goods.  Table 8 shows the distribution of panelists' bread color preferences. Compared to whole wheat bread, adding anchote starch in varying amounts to composite breads led to noticeable color differences, particularly in the crust. Bread with WAS_0_ had the highest number of panelists who strongly liked the color (21 out of 50), while bread with WAS_2_ had the fewest, indicating that the amount of anchote starch used influences the bread's color. The greatest dislike for color was seen in the composite bread with 90% wheat and 10% anchote starch, with one out of 50 panelists expressing strong dislike. In contrast, no panelists disliked the color of the other composite breads (WAS_0_, WAS_1_, WAS_3_, and WAS_4_), suggesting that adding anchote starch to wheat flour does not significantly alter the bread's color.

#### 3.7.2. Taste

The panelists evaluated the taste of the bread and summarized their findings in Table 8. The highest taste acceptability score of 12 “*extremely like*” ratings out of 50 panelists was given to bread made with WAS_0_ and WAS_4_, while the lowest score of 5 “*extremely like*” ratings was given to bread containing 95% wheat and 5% anchote starch. Only two panelists expressed strong dislike for the bread made with 80% wheat and 20% anchote starch, suggesting that the taste of the bread was not significantly affected by the proportion of anchote starch. Most panelists preferred the taste of bread made with 90% wheat and 10% anchote starch (WAS_2_) and 85% wheat and 15% anchote starch (WAS_3_).

#### 3.7.3. Odor

The panelists evaluated the odor of the bread, with their findings presented in Table 8. The highest “*extremely like*” rating was given to the bread containing 95% wheat and 5% anchote starch (WAS_0_), while the lowest “*extremely like*” rating was recorded for the bread containing 95% wheat and 5% anchote starch (WAS_3_). The bread made with 95% wheat and 5% anchote starch (WAS_1_) and 80% wheat and 20% anchote starch (WAS_4_) received the most disapproval, with one panelist out of 50 indicating that the addition of anchote starch did not affect the bread's odor.

#### 3.7.4. Texture

The panelists evaluated the texture (softness and chewiness) of the bread samples, with the results presented in Table 8. According to their responses, bread made with 100% wheat (WAS_0_) received the highest “*extremely like*” rating (19 out of 50), while bread with 90% wheat and 10% anchote starch received the lowest “*extremely like*” rating (eight out of 50 panelists). The bread made with 100% wheat (WAS_0_) also received moderate dislike. Mebpa et al. [22] noted that a harder crumb texture could result from higher fiber levels due to wheat bran substitution. Factors such as the condition of the bread components (e.g., fibers, carbohydrates, and proteins like gluten), whether damaged or undamaged, and the amount of water absorbed during dough mixing all influence the final texture of the product.

#### 3.7.5. Overall Acceptability

The panelists evaluated the overall acceptability of the bread loaves, with their findings presented in Table 8. The highest “*extremely like*” rating for overall acceptability was given to bread made with 100% wheat (WAS_0_), with 12 out of 50 panelists selecting it. The lowest “*extremely like*” ratings were observed for bread containing 95% wheat and 5% anchote starch (WAS_1_) and 90% wheat and 10% anchote starch (WAS_2_), with only seven panelists out of 50 selecting these options. A single “*highly dislike*” score was recorded for bread made entirely from wheat (WAS_0_), while 20 panelists rated WAS_3_ and 17 rated WAS_2_ as “*very much like*,” indicating that the addition of anchote starch to wheat flour was generally well-received. The sensory acceptability scores for breads made with a composite of wheat and anchote starch consistently remained above 6.00 on a 9-point scale, indicating various levels of preference. This suggests that anchote can be used to create acceptable loaves when blended with wheat flour up to a 20% proportion. The higher scores for wheat bread may be due to consumer familiarity with traditional wheat bread.

## 4. Conclusion

This study aimed to characterize the physicochemical properties of anchote starch and assess its effects on the nutritional profile, antinutritional compounds, and functional attributes when blended with wheat flour for bread production. The study revealed that anchote starch is a good source of carbohydrates, crude protein, and crude fiber, boasting significantly higher functional properties such as WAC, OAC, swelling power, and solubility index compared to other root crop flours. Substituting wheat flour with increasing levels of anchote starch led to an increase in carbohydrate content and a decrease in fat and fiber. Furthermore, adding anchote starch notably enhanced the mineral content, specifically calcium, iron, zinc, and sodium. While functional properties like swelling power and bulk density improved, water and OACs decreased. Importantly, anchote starch effectively reduced antinutritional factors such as phytate, cyanide, and tannin, although oxalate levels slightly rose. These findings demonstrate that blending anchote starch with wheat flour can produce composite flour and bread with desirable sensory qualities. Leveraging this underutilized starch not only enriches the mineral content of food products, benefiting human health, but also offers a promising avenue to boost nutritional value and mitigate the rising cost of exclusively wheat-based bread.

## Figures and Tables

**Table 1:** Blending composition of composite wheat–anchote starch.

**S.No.**	**Wheat flour (%)**	**Anchote starch (%)**	**Code**
1	100	0	WAS_0_
2	95	5	WAS_1_
3	90	10	WAS_2_
4	85	15	WAS_3_
5	80	20	WAS_4_

Abbreviation: WAS, wheat–anchote starch.

**Table 2:** Percentage yield and proximate composition of starch extracted from anchote.

**S. No.**	**Parameters (%)**	**Current study**	**Literature value**	**References**
1	Yield	70.41 ± 0.35	78.71 ± 0.07	[9]
2	Moisture	11.5 ± 0.5	9.36 ± 2.12	[10]
3	Ash	1.06 ± 0.06	1.08%	[9]
4	Fiber	0.14 ± 0.01	0.21% ± 0.06%	[10]
5	Fat	0.69 ± 0.05	0.75%	[25]
6	Protein	7.01 ± 0.49	7.0%–8.5%	[20]
7	Carbohydrate	79.60 ± 0.85	75%–90%	[26]
8	Gross energy (kcal)	356.54 ± 3.90	__	__

**Table 3:** Effect of wheat-to-anchote starch ratio on proximate composition of composite flour.

**% (wheat:starch)**	**Proximate composition (g/100 g DW)**						**Energy value (kcal)**
	**Moisture content**	**Total ash**	**Crude fat**	**Crude fiber**	**Crude protein**	**Carbohydrate**	
100:0	9.17 ± 0.29^a^	1.97 ± 0.01^a^	3.85 ± 0.02^b^	3.67 ± 0.03^a^	4.87 ± 0.36^a^	80.13 ± 0.21*b*^c^	374.71 ± 1.24^a^
95:5	9.50 ± 0.50^a^	1.99 ± 0.02^a^	3.98 ± 0.03^a^	3.33 ± 0.06^b^	4.81 ± 0.17^a^	79.71 ± 0.64^c^	373.97 ± 1.77^a^
90:10	9.73 ± 0.25^a^	1.99 ± 0.01^a^	3.76 ± 0.01^c^	3.02 ± 0.07^c^	3.89 ± 0.28^b^	80.64 ± 0.32^bc^	371.92 ± 1.05^ab^
85:15	9.55 ± 0.33^a^	1.93 ± 0.01^a^	3.62 ± 0.02^d^	2.16 ± 0.17^d^	3.80 ± 0.09^b^	81.1 ± 0.25^b^	372.18 ± 1.40^ab^
80:20	9.48 ± 0.03^a^	1.71 ± 0.04^b^	3.05 ± 0.01^e^	1.33 ± 0.03^e^	3.35 ± 0.24^b^	82.4 ± 0.24^a^	370.49 ± 0.25^b^
LSD	0.3531	0.22000	0.0537	0.2335	0.9933	0.9929	3.3546
CV (%)	3.53	1.22	0.55	3.22	6.15	0.46	0.34

*Note: *Means ± standard deviations with the same letter are not significantly different within a column at *p* < 0.05.

Abbreviations: CV, coefficient of variation to indicate the dispersion of data points around the mean in a series; LSD, least significant difference.

**Table 4:** Effect of wheat-to-anchote starch ratio on functional properties of composite flour.

**%(wheat:starch)**	**Functional properties of composite floor**			
	**WAC (%)**	**OAC (%)**	**BD (g/cm** ^**3**^ **)**	**SC (%)**
100:0	146.33 ± 3.51^a^	147.00 ± 4.00^a^	0.596 ± 0.00^e^	2.56 ± 0.02^e^
95:5	135.33 ± 6.51^ab^	130.67 ± 1.53^b^	0.606 ± 0.00^d^	2.90 ± 0.01^d^
90:10	129.33 ± 5.51^b^	123.33 ± 5.13^bc^	0.615 ± 0.00^c^	2.97 ± 0.01^c^
85:15	124.33 ± 0.58^b^	117.33 ± 4.62^c^	0.627 ± 0.00^a^	3.33 ± 0.02^b^
80:20	107.33 ± 3.79^c^	104.67 ± 4.16^d^	0.626 ± 0.00^b^	3.92 ± 0.02^a^
LSD	11.997	10.97	0.0001	0.0399
CV (%)	3.47	3.28	0.01	0.47

*Note: *Means ± standard deviations with the same letter are not significantly different within a column at *p* < 0.05.

Abbreviations: BD, bulk density; CV, coefficient of variation to indicate the dispersion of data points around the mean in a series; LSD, least significant difference; OAC, oil absorption capacity; SC, swelling capacity; WAC, water absorption capacity.

**Table 5:** Effect of wheat-to-anchote starch ratio on mineral composition of composite flour.

**%(wheat:starch)**	**Mineral composition (mg/1 g DW)**						
	**Na**	**Zn**	**Fe**	**Ca**	**Mn**	**Mg**	**K**
100:0	0.45 ± 00^d^	0.14 ± 0.01^e^	0.24 ± 0.01^c^	2.48 ± 0.12^e^	0.68 ± 0.05^a^	5.83 ± 0.10^a^	3.02 ± 0.18^a^
95:5	0.66 ± 0.01^c^	0.44 ± 0.02^d^	0.77 ± 0.09^b^	3.23 ± 0.01^d^	0.62 ± 0.02^a^	5.77 ± 0.09^ab^	2.41 ± 0.11^b^
90:10	1.12 ± 0.2^b^	0.88 ± 0.01^c^	0.85 ± 0.05^b^	4.15 ± 0.10^c^	0.52 ± 0.01^b^	5.56 ± 0.07^bc^	2.04 ± 0.11^c^
85:15	1.24 ± 0.03^b^	1.17 ± 0.06^b^	1.04 ± 0.07^b^	5.05 ± 0.06^b^	0.53 ± 0.01^b^	5.50 ± 0.06^c^	1.80 ± 0.11^c^
80:20	2.67 ± 0.13^a^	2.37 ± 0.19^a^	2.59 ± 0.21^a^	6.67 ± 0.13^a^	0.42 ± 0.00^c^	5.39 ± 0.10^c^	1.83 ± 0.09^c^
LSD	0.1641	0.2409	0.2912	0.2519	0.0616	0.2325	0.332
CV (%)	4.97	8.97	9.87	2.17	4.14	1.54	5.56

*Note: *Means ± standard deviations with the same letter are not significantly different within a column at *p* < 0.05.

Abbreviations: CV, coefficient of variation to indicate the dispersion of data points around the mean in a series; LSD, least significant difference.

**Table 6:** Effect of wheat-to-anchote starch ratio on antinutritional composition of composite flour.

**%(wheat:starch)**	**Antinutritional analysis (mg/1 g DW)**			
	**Oxalate**	**Cyanide**	**Phytate**	**Tannin**
100:0	2.20 ± 0.00^c^	3.16 ± 0.00^b^	1.23 ± 0.02^a^	0.03 ± 0.00
95:5	11.00 ± 2.20^b^	3.18 ± 0.03^b^	1.16 ± 0.02^b^	ND
90:10	13.93 ± 1.27^b^	3.21 ± 0.09^b^	1.18 ± 0.01^b^	ND
85:15	18.33 ± 2.54^a^	3.43 ± 0.08^ab^	1.04 ± 0.01^c^	ND
80:20	22.00 ± 0.00^a^	3.63 ± 0.20^a^	0.92 ± 0.01^d^	ND
LSD	4.3173	0.2823	0.043	
CV	11.91	3.17	1.45	

*Note: *Means ± standard deviations with the same letter are not significantly different within a column at *p* < 0.05.

Abbreviations: CV, coefficient of variation to indicate the dispersion of data points around the mean in a series; LSD, least significant difference; ND, not detected.

**Table 7:** Effect of blending ratio of anchote starch on the physical characteristics of the bread sample.

**% (wheat:starch)**	**Loaf weight (g)**	**Loaf volume (cm** ^**3**^ **)**	**Specific volume (cm** ^**3**^ **/g)**
100:0	81.20 ± 0.24^c^	360.88 ± 0.23^a^	4.44 ± 0.00^e^
95:5	83.80 ± 0.34^ab^	345.45 ± 0.53^b^	4.12 ± 0.00^d^
90:10	84.15 ± 0.21^b^	325 ± 0.13^c^	3.86 ± 0.00^c^
85:15	87.61 ± 0.58^b^	304 ± 0.62^d^	3.45 ± 0.00^b^
80:20	92.83 ± 0.63^a^	291.67 ± 0.16^e^	3.14 ± 0.00^a^
LSD	0.79	0.31	0.76
CV (%)	1.44	1.37	0.03

*Note: *Means ± standard deviations with the same letter are not significantly different within a column at *p* < 0.05.

Abbreviations: CV, coefficient of variation to indicate the dispersion of data points around the mean in a series; LSD, least significant difference.

**Table 8:** Effect of wheat-to-anchote starch ratio on sensory parameters of bread from composite flour.

**% (wheat:starch)**	**Color**	**Taste**	**Odor**	**Texture**	**Overall acceptability**
100:00	7.94 ± 1.57^a^	7.16 ± 1.79^a^	7.58 ± 1.59^a^	7.86 ± 1.50^a^	7.64 ± 1.24^a^
95:05	7.30 ± 1.61^a^	6.84 ± 1.61^b^	7.08 ± 1.54^ab^	7.00 ± 1.75^ab^	7.30 ± 1.05^a^
90:10	7.40 ± 1.43^a^	7.18 ± 1.30^a^	7.38 ± 1.29^a^	7.54 ± 1.18^a^	7.42 ± 1.01^a^
85:15	7.40 ± 1.09^a^	7.34 ± 1.38^a^	7.54 ± 1.47^a^	7.28 ± 1.75^a^	7.42 ± 1.16^a^
80:20	7.40 ± 1.59^a^	7.06 ± 1.94^a^	7.06 ± 1.70^ab^	7.00 ± 1.78^ab^	7.10 ± 1.36^ab^

*Note:* Response of 50 panelists in mean ± standard deviation.

## Data Availability

The data that support the findings of this study are available on request from the corresponding author. The data are not publicly available due to privacy or ethical restrictions.
